# Using Flexible Curved Noncontact Active Electrodes to Monitor Long-Term Heart Rate Variability

**DOI:** 10.1155/2020/8867712

**Published:** 2020-07-08

**Authors:** Ji-Jer Huang, Zhe-Lin Cai

**Affiliations:** Department of Electrical Engineering, Southern Taiwan University of Science and Technology, Tainan City 71005, Taiwan

## Abstract

The purpose of this study is to utilize flexible curved noncontact active electrodes to develop a nonperception, long-term, and wireless heart rate monitoring system. This study also verified the functions and capabilities of the system and provided information on physiological parameters recorded during our tests. Our system was used in tandem with a commercially standard measurement system; both systems were used to measure ECG signals on 10 healthy subjects under the simulated home and office scenarios. We verified the R-peak measurement accuracy of our system and used *T*-tests to analyze the data collected by both systems; our system reached an average sensitivity value of 0.983 and an average positive predictive value of 0.991 over several different scenarios where R-peak measurements were also highly accurate. The R-R time intervals of our system were highly consistent with the standard system. The correlation coefficient calculated reached almost one, and the differences between the two systems mostly fell within the ±10 ms range. Further study of the HRV time-domain parameters under four different scenarios showed no significant differences in most HRV parameters compared to the measurements by the standard system. We also used our system to record long-term heart rate signals.

## 1. Introduction

Adapting to lifestyle changes and the rapid pace of today's society can be stressful for our lives. Over a long period of time, the stress may cause cardiovascular disorders. Additionally, the emergence of cancer and related illnesses are also closely correlated with stress. We often do not notice when we are under stress. Therefore, it is difficult for people to determine whether or not they are suffering from too much stress. Many people may not be aware that stress can cause symptoms such as constipation, poor sleep, allergies, fatigue, anxiety, and weight gain, all of which are harbingers of serious physical disorders. Furthermore, excessive stress can result in physical imbalances that affect the autonomic nervous system (ANS) and the endocrine system. We hope that continuous measurements of heart rate signals and calculations of the heart rate variability (HRV) parameters can help us assess the status of the sympathetic and the parasympathetic nervous systems. Those HRV parameters can also serve as important indicators of the stress level. HRV is correlated with physical and mental health and can also reflect the state of balance between the autonomic and nonautonomic nervous systems. However, the process of taking physiological measurements can be easily impacted by environmental factors, such as tangled wires and electrode contacts, which may cause discomfort in patients. Also, the “white coat effect” of hospitals has been well known to affect the measurements of physiological signals in patients.

Studies of skin-electrode interfaces in the literature report that bioelectrical electrodes mostly fall into one of three categories, depending on the type of contact medium used. Wet electrodes are the ones most commonly seen in clinical and experimental settings. These electrodes conduct electricity through electrolyte gels spread over the skin. The gels may cause skin irritation and patient discomfort. Possible allergic reactions to the gel, inconvenience of use, and shorter electrode lifespans are the main drawbacks of wet electrodes [1]. By contrast, dry electrodes, defined by Searle et al. and Chi et al. as electrodes that come into direct contact with human skin [2, 3], are placed on the stratum corneum (SC), which has high resistivity. Dry electrodes rely on conductive materials in the environment or on the body (for example, sweat or water) to conduct electricity, and therefore, the electrical coupling abilities of dry electrodes may change over time due to differences in available conductive materials. Finally, the third type of electrode, the capacitive electrode [4, 5], is one that does not come into direct contact with the skin and contains an isolation layer made of nonconductive material such as human hair, cloth, clothing, or air. The main feature of capacitive electrodes is that they are nonconductive electrodes that do not come into electrical contact with the human body. They do not rely on liquid media to conduct electricity. However, unlike wet electrodes, capacitive electrodes measure displacement currents and not electric charges flowing through a skin-electrode interface. Because of this, capacitive electrodes are safer to use and require neither skin exfoliation nor the use of conductive gels. They are ideal for use in long-term patient monitoring, especially in situations where there are no medical personnel available. In 1967, Richardson pioneered the research of using capacitive electrodes to make physiological measurements; he used capacitive electrodes coated with a thin, nonconductive layer of aluminum oxide to measure bioelectrical signals [6]. Noninvasive capacitive electrodes use nonconductive materials to form a layer of insulation between the electrode and the human body, resulting in high impedance levels of several dozen pF in capacitance and several hundred MΩ in resistance [7], which makes them safer for users and prolongs their useful lifespan. However, an obvious drawback of capacitive electrodes is that the signals captured tend to be weaker.

In the literature, many researchers have pointed out that there are three types of interference that are present when capacitive electrodes are used to capture ECG signals. The first type of interference comes from electromagnetic fields in the measurement environment, the second type from friction on the skin-electrode interface, and the third type, from changes in capacitance coupling caused by vertical movements on the interface when there is a direct current running through the two ends of the coupled capacitor. Earlier measurement systems utilized capacitive grounding techniques to reduce common-mode noise in power lines or other electronic devices. Although this reduced impedance between the human body and the ground, levels of common-mode noise were still high and often caused a saturation effect when signals entered electrodes. Some researchers used a driven-right-leg circuit for capacitive electrodes and used common-mode feedback to reduce common-mode interference [8, 9]. Electrode coupling capacitance levels are dependent on the distance between the electrode surface and the skin. Movement perpendicular to the skin-electrode interface induces a change in coupling capacitance values, which then produces low-frequency noise caused by voltage-measured fluctuations. Researchers discovered that the elasticity of soft electrodes stabilized contact between an electrode and the human body and minimized motion noise [10]. An alternative suggested solution was a wearable device worn close to the skin, such as a belt or a skintight vest, which can help to inhibit noise generated by vertical motions. Horizontal movements are another source of motion noise. Many researchers have studied the phenomenon and mechanism of noise made by motion friction and have used highly elastic soft electrodes to minimize signal changes caused by friction [11].

Additionally, the direct voltage running through the two ends of the coupled capacitor come from different sources, with one end being a bias current from an amplifier and the other being electric charges generated by electrodes rubbing against the skin. It is necessary to provide a biased pathway for the direct currents, as flowing it through the high-resistance back-end and high-gain circuits will create signal saturation and make it impossible to capture physiological signals. Biased pathways are commonly set up with a resistor that has an impedance level higher than that of the amplifier input impedance. The resistor is connected to the input end and the power ground of the amplifier. The amplifier input impedance is thus on a common node with the biasing resistor. Past literature has proposed many methods to provide the biased current with the necessary pathway without affecting the high input impedance of the front-end amplifier, for example, the use of bootstrapping, which uses feedback to drive the voltage at the input terminals and minimize signal leakage to the biased current [12, 13]. Alternatively, utilization of internal amplifier bias may result in low-frequency drift; more commonly, the amplifier output terminal is connected to a circuit that filters out the direct current [14–16]. Some researchers have pointed out that the impedance level of the biased current pathway does not need to be a precise value and that saturation of the front-end circuit can be prevented as impedance levels meet threshold values. This impedance may cause an increase in measured voltages signals, but it can be removed through back-end filtering or negative feedback.

Furthermore, the coupling capacitance of the skin-electrode interface is equivalent to the stray capacitance of the electrode when noncontact electrodes are used to take subject measurements, and this can cause signal attenuation. To obtain high-quality signals, we must reduce this stray capacitance and issues related to the biased current, as both of these may reduce the input impedance of signals. For this reason, guarding and neutralization techniques are often applied to avoid the effects of stray input capacitance from the amplifier and to minimize the input capacitance of the circuit [13, 17]. Capacitive electrodes make it possible to capture bioelectrical signals even when clothing is worn. There have been a number of studies on the measurement quality due to wearing different clothing [18–20] or using different accessories such as beds [21], chairs [22], bathtubs [23], wheelchairs [24], and on car seats [25, 26]. However, many of these studies have only tested the feasibility of the technology and not the long-term monitoring or recording functions of these systems.

HRV is calculated by measuring changes in the intervals between heartbeats, as well as increases and decreases in heart rate, due to the effects of sympathetic mediators (adrenalin and norepinephrine) and parasympathetic mediators (acetylcholine) on the sinoatrial and atrioventricular nodes [27]. HRV is an important indicator of cardiovascular autonomy [28], as well as mental and physiological health. Some researchers have suggested that HRV can be used to reflect changes in emotional shifts [29]. Many researchers have proposed some mathematical and statistical models for HRV calculations. For example, in 1996, the European Society of Cardiology and the North American Society of Pacing and Electrophysiology published a paper listing international standards for clinical application of HRV measurements and physiological indications that can be gleaned from HRV figures [28]. These international standards contain standardized methods for analyzing HRV. The two most common parameters used are the time-domain parameter and the frequency-domain parameter. Additionally, it was proposed that two SDNN variables (the SDNN index and SDANN index) are calculated by breaking down 24-hour monitoring periods into 5-minute subintervals. It is recommended that each continuous section of recording time should be twice that of the minimum signal period [30] for the calculations of frequency-domain parameters. Many researchers suggest a minimum duration of 5 minutes be used for short-term HRV measurements [30–32].

The purpose of this study is to develop a capacitive heart rate measurement system that is noncontact, nonbinding, and nonperception and can be used for long-term measurements through the utilization of noncontact active electrodes. In our system, the guarded circuits and biased-path pathways were designed. The curved noncontact active electrodes are placed in the back of a commercially available adjustable chair commonly used in the home and office. The curved electrodes and ergonomically designed chair back make it possible for our system to adapt to different body shapes and different types of chairs, bringing the electrodes as close as possible to the skin of the subject, so the subject's heart rate can be measured. At this time, the subject does not perceive the presence of these electrodes. Since the system does not come into contact with the skin and the subject's movements are not limited by hardware or wires, there is no need for subjects to cease their daily activities when measurements are being taken. This can obviate the mental and physiological effects associated with more formal measurement settings. Therefore, the system can be used for long-term monitoring.

## 2. Materials and Methods

### 2.1. System Structure

**Figure 1:**
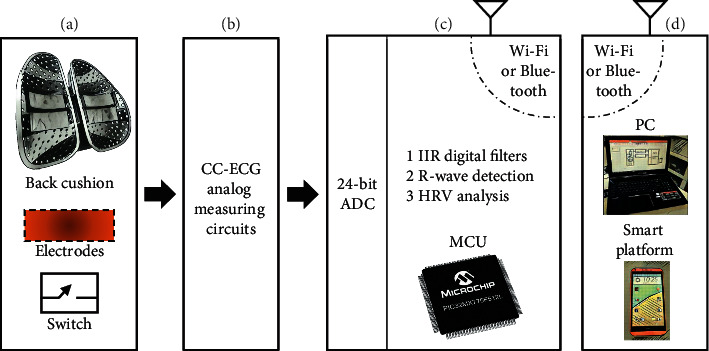
System structure.

This paper presents a capacitive coupling-electrocardiogram (CC-ECG) measurement system using noncontact active electrodes. In the system structure, Figure 1(a) shows the microswitch in the chair back for the activation of the system and the capacitive coupling electrodes used to measure ECG signals. These signals are sent to the CC-ECG measurement circuit (as shown in Figure 1(b)) and digitally converted by a 24-bit ADC converter before being sent to the MCU chip, a 32-bit microcontroller manufactured by Microchip (serial number PIC32 M × 775 F512L), as shown in Figure 1(c). The digital signals were then filtered through an infinite impulse response IIR Butterworth filter before R-peak detection because the Butterworth filter has better flat features. The HRV parameters are then calculated within the time intervals between R waves. These parameter values are then transmitted via the Wi-Fi interface provided by the Microchip RN1723 module (Figure 1(d)). Our system also supports the use of Bluetooth transmission of these parameter values to the information consolidation platforms, which consist of remote computers or smart devices. Once received, physiological parameter values are stored, analyzed, and displayed on the platform for future application.

### 2.2. Design of Capacitive Coupling Electrodes

**Figure 2:**
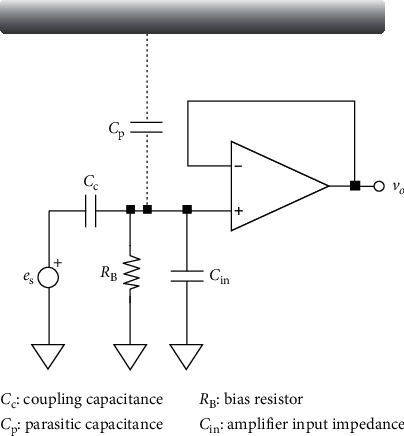
Simplified diagram of the front-end circuit.

The front end of the capacitive coupling electrodes consists of voltage followers in which the amplifier used is an OPA121 chip manufactured by Texas Instruments with an input impedance of Zin = 10^13^Ω || 1 pF. As shown in Figure 2, the operational amplifier is used as a voltage follower. Better circuit performance can be obtained according to decreased coupling capacitance (*C*_c_) and parasitic capacitance (*C*_p_), and therefore, it is necessary to include an amplifier with high input impedance. Apart from using the shielding effect to reduce the effects of stray capacitance (*C*_shield_), it is also necessary to provide a pathway for the biased current using a high-impedance biased *R*_B_ resistor to increase thermal noise. The input impedance of the *R*_B_ resistor and the input capacitor *C*_in_ needs to be much higher than that of *C*_c_ to avoid weakening captured signals. *C*_c_ and *C*_in_ are capacitive dividers, so the input capacitance of *C*_in_ needs to be low to avoid causing voltage division and weakening of inhibition capabilities toward interference and motion noise. Overall, biased currents with high impedance and low input capacitance are both keys to lowering noise.

To form the biased current pathway, a high-impedance resistor is connected to the ground from the input terminal of the electrode, thus forming a simple but stable direct biased pathway. The biased resistor used in our system has an impedance of 150 GΩ. The internal capacitance of the voltage follower is 1 pF; we only looked at R waves in incoming ECG signals when considering the frequency of incoming signals (the frequency of R waves is around 10 Hz), making the equivalent impedance around 15 GΩ. Thus, the impedance of the biased pathway is around ten times that of the voltage follower, and the internal input capacitance of the voltage follower becomes the dominant impedance. For low-frequency noise from direct currents, our biased impedance will dominate input impedance, and therefore, increases in biased impedance would have helped inhibit direct current noise. However, because our system is used to measure heart rate variability, we considered the biased impedance pathway provided by the 150 GΩ impedance to be suitable for our purposes.

**Figure 3:**
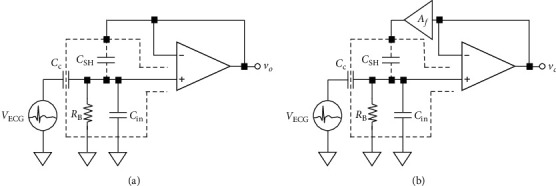
(a) Active guarded circuit. (b) Positive feedback gain loop.

To decrease the input capacitance, we use guarded currents to enhance signal quality and to decrease the noise caused by the high impedance of the voltage follower, other currents, and environmental factors. The input terminal is wrapped in insulating metal (as shown in Figure 3(a)), causing the input terminal and the insulating metal to form stray capacitance *C*_shield_. In order to ensure that this stray capacitance pathway *C*_shield_ would not cause noise within our measurement space and affect our input terminal, we equalized the electrical potential at both terminals and connected the input terminal of the voltage follower to the insulating metal terminal to form a feedback driving circuit that is equal to input signals. This connection decreased the effect of noise caused by stray capacitance and achieved the objective of preventing electromagnetic interference. Figure 3(a) shows a circuit with a feedback gain of 1, which may cause the circuit to oscillate. To avoid oscillation, we set the Af gain to 0.9, as shown in Figure 3(b), based on current research by other scholars [33]. Because of this gain setting, the best measurement results can be obtained.

### 2.3. Flexible Curved Electrodes and CC-ECG Measurement Circuit

**Figure 4:**
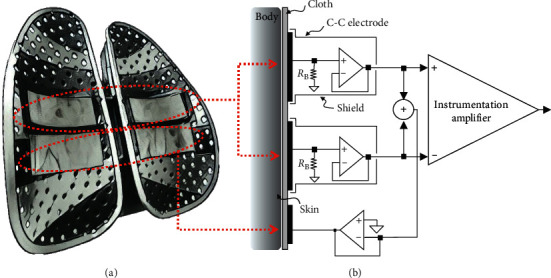
(a) Electrode positions on chair back. (b) The front-end circuit.

In this system, we use four stainless steel sheets with flexible and curved surfaces as electrodes. The length and width of the electrode are 5 cm × 3 cm and the thickness is 0.3 mm. An electrode with a flexible and curved surface can achieve better adhesion to the body surface of the subject so that the electrical signal of the subject can be more easily captured. The material used in stainless steel is mainly considered to be less prone to oxidation over long-term use. We placed two capacitive coupled electrodes (above) and two reference electrodes (below) into a commercially available adjustable chair back, as shown in Figure 4(a). The position of our reference electrode is different from that of most other studies; researchers generally placed their reference electrodes at the bottom of chair cushions where it would be close to the buttock area of subjects. However, it was difficult for us to place an additional electrode at the buttock area of our chair back as this would have required additional wires and devices, so we chose to place our reference electrode beneath the capacitive coupled electrodes. The noncontact reference electrode and the front-end circuit are connected in a driven-right-leg manner, as shown in Figure 4(b). A microswitch is also placed in the chair back. When a user's back slightly touches the chair back, the change in the electrical potential of the microswitch is transmitted along the circuit to the MCU, and the system is then activated. In the same way, the system is shut down automatically when the user vacates the chair.

**Figure 5:**
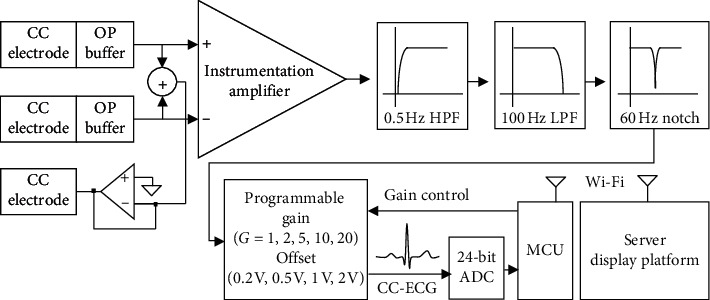
Diagram of the CC-ECG measurement circuit.

The capacitive ECG measurement circuit serves as the front-end circuit of the electrodes with the primary purpose to inhibit noise so that physiological signals can be captured and amplified without loss of quality.  Figure 5 shows a diagram of the capacitive ECG measurement circuit. Signals captured by the capacitive coupled electrodes are passed through an amplifier with a high common-mode rejection ratio (CMRR), where the differential between the two electrodes is obtained. This is equivalent to the Lead I signal in standard ECG measurements. The signals are then run through a circuit composed of a 0.5 Hz high-pass filter (HPF), a 100 Hz low-pass filter (LPF), and a 60 Hz band-stop filter before they pass through a programmable gain-adjusted circuit. When the system detects that the signal is too small (<50%) or too large (>75%), the system will perform gain adjustment to increase or decrease the gain of the analog circuit to obtain a better signal. The output signals are then run through a 24-bit ADC converter for digital conversion, and the digital information is transmitted to the MCU via an SPI interface. The sampling frequency of the ADC is set to 1 kHz.

We implement digital filters to obtain better-quality signals and minimize noise effects. Appropriate use of digital filters can effectively decrease the cost of analog circuits and make future adjustments easier to manage. We also use 0.5 Hz HPF and 100 Hz LPF IIR Butterworth filters in this study to install on MCUs. The order number of two IIR Butterworth filters are equal to 9.

The MCU transmitted processed physiological parameter values and other information through an RN1723 Wi-Fi module and a server using a TCP/IP protocol. RN1723 is an independent embeddable 802.11 b/g Wireless Local Area Network (WLAN) module. The system is set up using the commands via UART. Low power consumption is one of the main features of the RN1723 module, making it suitable for use in applications that need to be connected via Wi-Fi for a long period of time, such as in physical monitoring systems using various types of sensors. After the connection between our system and routers is established through our Wi-Fi interface, our system is able to transfer data back to our client server remotely.

### 2.4. R-Peak Detection and Calculations

**Figure 6:**
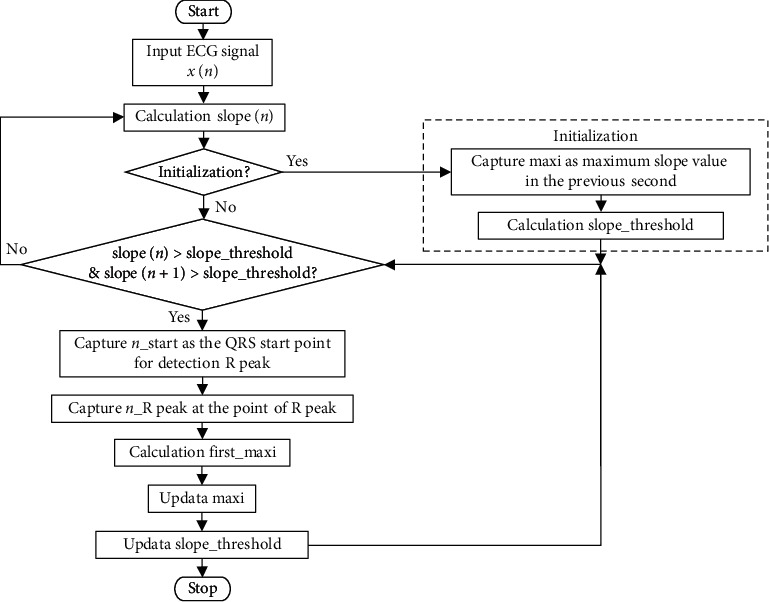
The flow diagram of detecting R-peak process.

In this study, our algorithm is used to detect R-peaks [34]; the detection processing is outlined in Figure 6. Once ECG signals are captured, this algorithm calculates the slope of each sample point preceding and following the ECG sampling point *x*(*n*) for the time domain, as shown in equation (1). This equation carries a weighted value to highlight changes in slope. Before we explain how the slope threshold is calculated, let us first explain how the parameter for the maximum slope value (maxi) is calculated. The initial value of the maxi parameter is the maximum value of slope (*n*) taken within one second after sampling. Newly detected R-peak waves then update this value. The algorithm for calculating the slope threshold (slope_threshold) is as shown in equation (2). Furthermore, we found that when the slope (*n*) of two continuous points both exceeded the dynamic slope threshold, those points could then serve as the start of the QRS wave and be further extended to find the maximum value, which is defined as the peak of the R wave. After this, each detected wave peak is used to update the maxi parameter and the slope_threshold parameter, as shown in equations (2) and (3). The first maxi parameter is obtained via equation (4).

(1) $$\begin{array}{c} \text{slope}\left(n\right)=-2x\left(n-2\right)-x\left(n-1\right)+x\left(n+1\right)+2x\left(n+2\right), \end{array}$$

(2) $$\begin{array}{c} \text{slope}\_\text{threshold}=\frac{\text{maxi}}{2}, \end{array}$$

(3) $$\begin{array}{c} \text{maxi}=\frac{\text{first}\_\text{maxi}-\text{maxi}}{16}+\text{maxi,} \end{array}$$

(4) $$\begin{array}{c} \text{first}\_\text{maxi}={\text{height}}_{n\_R\_\text{point}}-{\text{height}}_{n\_\text{start}\_\text{QRS}}\text{.} \end{array}$$

### 2.5. Heart Rate Variability

The method used for analyzing heart rate variability in this study is done by the HRV international standards [35] published by the European Society of Cardiology and the North American Society of Pacing and Electrophysiology. Time-domain analysis is conducted on a series of signals in continuous R-R time intervals. Measurements are conducted on the average or relative average heart rate in the R-R time interval, as shown in equation (5). The standard deviation of the R-R time interval (SDNN) is defined as shown in equation (6), with RR_*j*_ representing the value of the *j*th R-R time interval and N being the total number of continuous intervals. SDNN reflects the overall change in the R-R time interval sequence, and the root means square of the continuous R-R time interval difference (RMSSD) is as shown in equation (7). The coefficient of variation (CV) of the R-R time interval is as shown in equation (8), where the continuous R-R time interval difference is used to derive another indicator (NN50), namely, the number of continuous time interval differences that exceed 50 ms, as shown in equation (9). Time-domain analysis is easier to implement in portable systems.

Our noncontact monitoring system does not interfere with the daily lives of users while our back-end display platform automatically captures and records data. Physiological signals are transmitted to remote computers and human-machine interfaces through the Wi-Fi interface. The human-machine interface used in this system is based on the LabWindows software developed by US Company NI. The display interface mainly displays the HRV parameter values of users seated against the chair back; parameters shown include HR, mean RR, SDNN, RMSSD, CV, NN50, and pNN50. ECG waves captured by the system in real time are also displayed. This system makes it possible to capture information and store them in personalized databases to facilitate continuous monitoring of individuals over a long period of time.

(5) $$\begin{array}{c} \text{Mean RR}=\frac{\sum_{j=1}^{N}{\text{RR}}_{j}}{N}, \end{array}$$

(6) $$\begin{array}{c} \text{SDNN}=\sqrt{\frac{1}{N-1}\sum_{j=1}^{N}{\left({\text{RR}}_{j}-\bar{\text{RR}}\right)}^{2}}, \end{array}$$

(7) $$\begin{array}{c} \text{RMSSD}=\sqrt{\frac{1}{N-1}\sum_{j=1}^{N-1}{\left({\text{RR}}_{j+1}-{\text{RR}}_{j}\right)}^{2}}, \end{array}$$

(8) $$\begin{array}{c} \text{CV}=\frac{\text{SDNN}}{\mathrm{mean}\;RR}\cdot 100\%, \end{array}$$

(9) $$\begin{array}{c} \mathrm{pNN}\;50=\frac{NN50}{N-1}\cdot 100\%. \end{array}$$

## 3. Experimental Procedures

### 3.1. Experiments under Different Scenarios and Tests over a Long Period of Time

**Figure 7:**
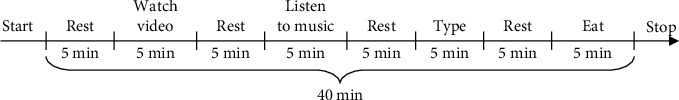
Scenario process diagram.

We conducted several experiments to test our capacitive heart rate measurement system and its performance in different daily life scenarios, as shown in the process flow diagram of Figure 7. We conducted continuous measurements lasting 40 minutes. In addition to peaceful activities such as video-watching and music-listening, we also included dynamic daily activities such as typing and eating for a total of four scenarios. During our tests, we asked ten subjects (age: 23.5 ± 5.2 years, height: 172.3 ± 10.2 cm, weight: 69.2 ± 15.6 kg, male: 7 people, and female: 3 people) to sit against our chair-back system and run through the various scenarios while we simultaneously recorded their CC-ECG and standard ECG. Those subjects who did not have any age limit and cardiovascular disease were chosen for our tests, but in consideration of technical limitations and daily usage scenarios, subjects dressed in overly bulky clothing were asked to remove their clothing in order for the test to run smoothly.

Another experiment was focused on measurements taken over a long period of time. This experiment was conducted on graduate students who spent an extensive amount of time on their computers. Measurements were taken continuously from 9 am to 5 pm over 8 hours. The graduate student entered the lab at 9 am. Excluding the time period when they left to purchase food, eat, or go to the bathroom, they spent pretty much their whole day doing work at their computers until they left the lab at 5 pm.

### 3.2. Verification of System Structure

**Figure 8:**
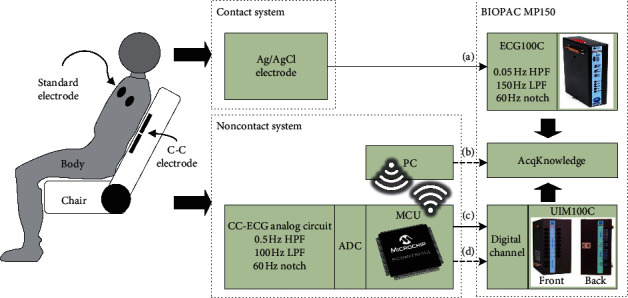
System connection structure.

**Figure 9:**
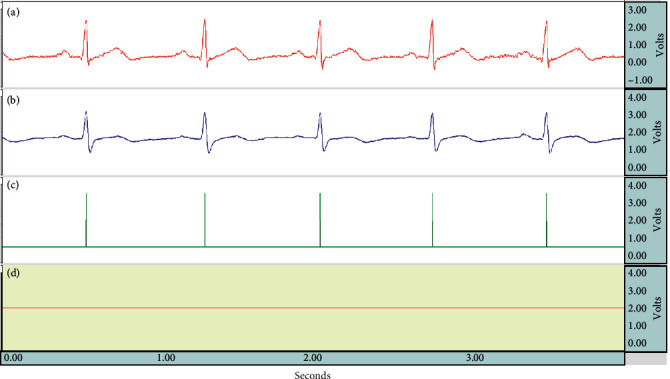
(a) ECG100C. (b) CC-ECG. (c) R peak. (d) Sit/leave.

We exercise a number of experiments to assess the performance of our capacitive heart rate system which can be used on computer chairs at home or in office environments and to verify the heart rate measured by the capacitive system using ECG signals measured by standard measurement tools. We used the AcqKnowledge software and the BioPAC MP150 multichannel physiological measurement system to form a platform for data capture, analysis, and storage. The BioPAC MP150 measurement system is composed of an MP150 device, a UIM100C universal interface module, and an ECG100C ECG amplifier module. After careful consideration of how our experiments should be structured, we decided to use Ag-AgCl electrodes and directly stuck them onto the chests and calves of our subjects instead of placing them on the limbs. We used the ECG100C ECG amplifier module to perform simultaneous Lead I contact measurements, while our subjects leaned back against the noncontact active-electrode chair back. One experiment lasted 40 minutes, and another one lasted 8 hours in order to test the long-term measurement performance of our system. The ADC filter parameter of the ECG100C ECG amplifier module was set to a high-pass cutoff frequency of 0.05 Hz and a low-pass cutoff frequency of 150 Hz, while the band-stop frequency was set to 60 Hz. The UIM100C universal interface module was connected to the MP150 through an external connection to provide 16 channels of digital input (front panel) and 16 channels of analog input (back panel). The system connection structure is shown in Figure 8. We used the touch function of the AcqKnowledge software to verify different input signals and captured and conducted simultaneous capture, collection, and analysis of signals.  Figure 9 shows the waves captured by our system.

### 3.3. Data Analysis and Verification

We evaluated the quality of our signal measurement through sensitivity (Se) and positive predictive value (PPV) analyses. The analyses were divided into three parts: the first part was verification of R-peak detection accuracy in determining the stability of the system; the second part was verification of R-R time intervals and analysis of correlation with signals measured by the BioPAC MP150 (ECG100C); the third part was verification of differences in HRV parameters, where we took R-R time interval information captured from our 10 test subjects and used our self-developed software to calculate HRV parameters for comparison.

To verify the accuracy of R-peak detection, we obtained standard ECG waveform detection results by entering signals captured by the MP150 to the BIOPAC AcqKnowledge software. The R-peak detection is run under the LabWindows environment to detect the R-peak. We first used the software to compare the relative positions of R-peaks. Researchers conducted additional interpretation when abnormalities were detected. Based on our detection analysis, we divided R-peak detection results into four categories: true positive (TP), where there was an actual R-peak, and our system detected the R-peak; false positive (FP), where there was no actual R-peak, but our system detected an R-peak; false negative (FN), where there was an actual R-peak, but the system did not detect an R-peak; and true negative (TN), where there was no R-peak and the system did not detect an R-peak. It is difficult to calculate TN in such a detection scheme, so this is likely the reason TN is not used in the performance metrics.

After we had categorized all our R-peak detection results, we used Se and PPV parameters to assess the accuracy of R-peak detection, as shown in equations (10) and (11). The Se parameter refers to the percentage of R-peaks detected by our system as compared to the actual amount of R-peaks. Higher sensitivity indicates higher R-peak detection accuracy. The PPV parameter refers to the percentage of actual R-peaks among the R-peaks detected by our system; this parameter was used to measure the error rate of the system. Higher PPVs indicate higher R-peak detection accuracy.

(10) $$\begin{array}{c} \text{Sensitivity}\left(\text{Se}\right)=\frac{\text{TP}}{\text{TP}+\text{FN}}, \end{array}$$

(11) $$\begin{array}{c} \text{Positive predictive value}\left(\text{PPV}\right)=\frac{\text{TP}}{\text{TP}+\text{FP}}. \end{array}$$

According to the range of the R-R time interval, if the heart rhythm exceeds 1500 mm·sec (40 bpm) and less than 400 mm·sec (150 bpm), it will be regarded as an erroneous heart rhythm signal that will be removed. In that case, we referred to the standard ECG measurement waveform to get the correct false and positive information. After removing false positive and false negative, we analyzed disparities in HRV values by entering R-R time interval information into our parameter algorithms for comparative analysis. We also used the algorithms to calculate respective HRV parameters for HRV_CC-ECG_ and HRV_Standard_ under different scenarios. Correlation and differential analysis were then conducted on HRV_CC-ECG_ and HRV_Standard_ for the same scenario. All analyses were conducted using the SPSS (version 20) software developed by IBM, with confidence intervals set to 99% and significance levels set as smaller than 0.01 (*p* < 0.01).

## 4. Results

### 4.1. Noncontact Chair-Back Capacitive Heart Rate Measurement System

**Figure 10:**
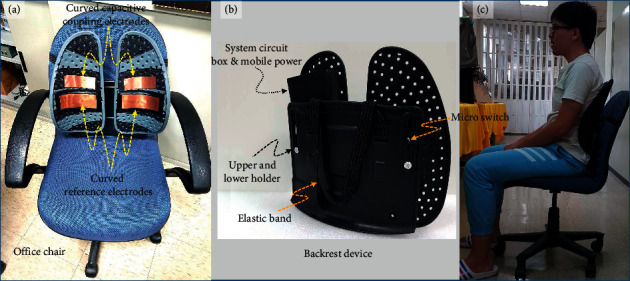
(a) Measurement system and office chair. (b) Chair-back device. (c) Actual measurement conditions.

**Figure 11:**
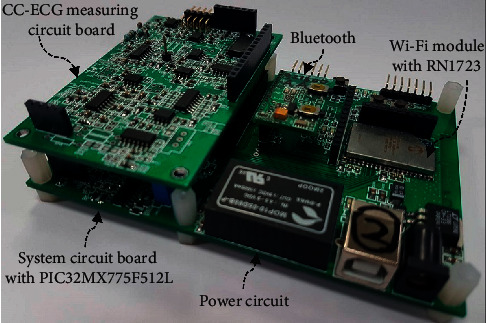
Image of the actual system circuit.

We used a noncontact measurement technique that does not interfere with the daily lives of users to establish a nonperception physiological measurement system, as shown in Figures 10(a) and 10(b). We used a commercially available adjustable chair back as the basis for our system. An ergonomically designed chair back was chosen to decrease the gap between the chair back and the back of human users and also to make it possible to adjust the chair back in accordance with user body shapes. Curved capacitive coupled electrodes are suitable for adaption to different body curvatures and make it easier for users to press their backs against the back of the chair, thus enhancing the signal-capturing ability of the system. The use of a chair back widens the field of application and makes it more convenient to use as the system does not have to be limited to a single office chair. As can be seen from Figure 10(c), this chair-back device can be placed on any household chair. The integrated circuit box and power supply (provided by a power bank) are placed inside the chair back so that the chair could be moved freely.  Figure 11 shows the circuit board of the integrated system circuit box. The circuit board is composed of a 32-bit microcontroller paired with a Wi-Fi module (RN1723). This circuit board also utilizes separate digital and analog circuit modules where the lower circuit board is used for control, transmission, and power switch of digital information and the upper circuit board is used to capture analog CC-ECG signals that are digitally converted by a 24-bit, high-resolution ADC, allowing for better tolerance of biased DC voltages. A Wi-Fi interface is used for data transmission. Power is provided by a commercially available 5V power bank, which is rechargeable and thus more convenient for use. The system also includes a self-developed user interface that ran on the human-machine interface.

**Figure 12:**
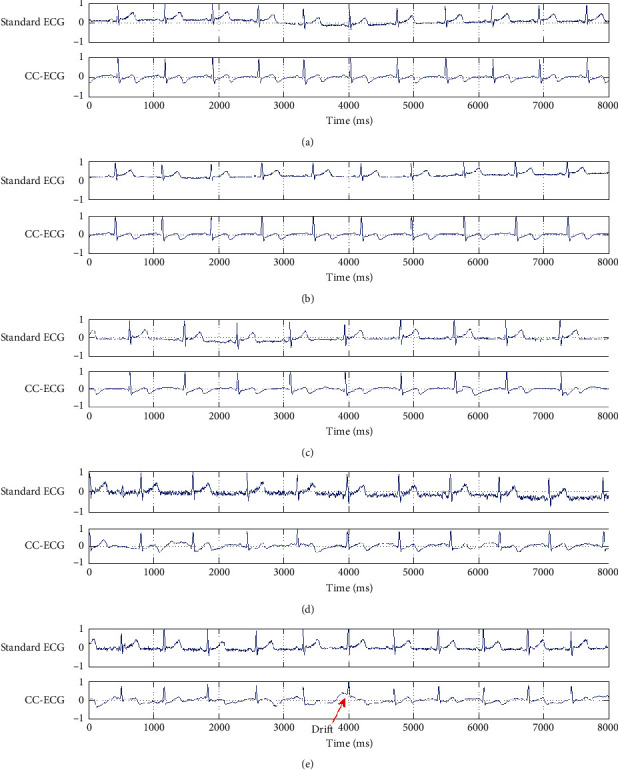
Standard and coupling capacitance ECG under (a) resting, (b) video-watching, (c)music-listening, (d) typing, and (e) eating.

We simultaneously used our system and a commercially available instrument to obtain ECG graphs, as shown in Figure 12. The graphs show standard ECG charts captured by the commercial instrument (BioPAC MP150) and the others captured by our capacitive coupled electrode system under resting, video-watching, music-listening, typing, and eating scenarios. We can see from Figures 12(d) (typing) and 12(e) (eating) that low-intensity EMG signals can be captured by BioPAC MP150 under these scenarios. Additionally, in Figure 12(e) (eating), we can see that there is some drift in the waves produced by the capacitive coupled electrode system. The system is common in capacitive coupled electrode systems, mainly due to changes in the distance between the body and the electrodes.

### 4.2. Accuracy of R-Peak

**Figure 13:**
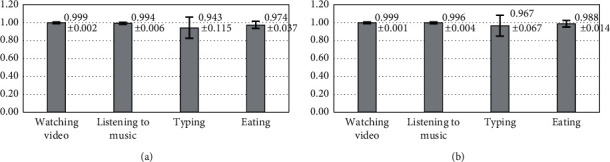
(a) Sensitivity (Se) and (b) positive predictive values (PPV) for the ten subjects under different scenarios.

Our ten subjects underwent four different scenarios in 40 minutes. The results are shown in Table 1. From left to right, the columns list the number assigned to each subject, their actual heart rate, their detected heart rate, and then their TP, FP, FN, Se, and PPV results. Average sensitivity was 0.983, and average PPV was 0.991.  Figure 13(a) shows the sensitivity under different scenarios, and Figure 13(b) shows the PPV results. We can see that more dynamic behaviors, such as typing and eating, produced slightly lower Se and PPV results. Se values stayed above 0.943 and PPVs stayed above 0.967. From this analysis, we can see that the system has a fairly accurate R-peak detection ability.

### 4.3. RR Time Interval Association

**Figure 14:**
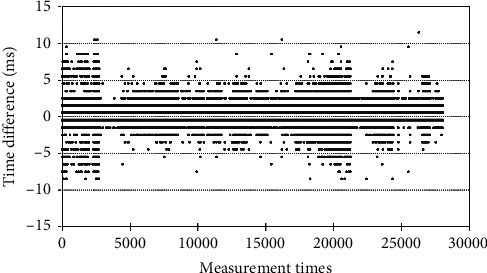
Differences in R-R time intervals.

Following the R-peak verification to detect false positives and false negatives, according to the reasonable range of the R-R time interval, we removed abnormal values and used the SPSS software to conduct correlation analysis on R-R time intervals. We used a scatter plot to observe the relationship between standard measurements and the R-R time intervals captured by our system. We can see from the figure that the two have a very high positive correlation. The Pearson correlation coefficient *r* for the two sets of data was 1.00, and the coefficient of determination (*r*^2^) was close to 1. We also conducted a differential analysis of the two sets of data to determine the differences between the two, as shown in Figure 14. We can see that most time differences fell within the ±10 ms range and that when 85 beats/min was set as one cycle, the difference only fell within the ±1.4% range. This indicates that the R-R time intervals obtained by our system are highly consistent with standard data captured by commercial instruments.

### 4.4. HRV Differences

**Figure 15:**
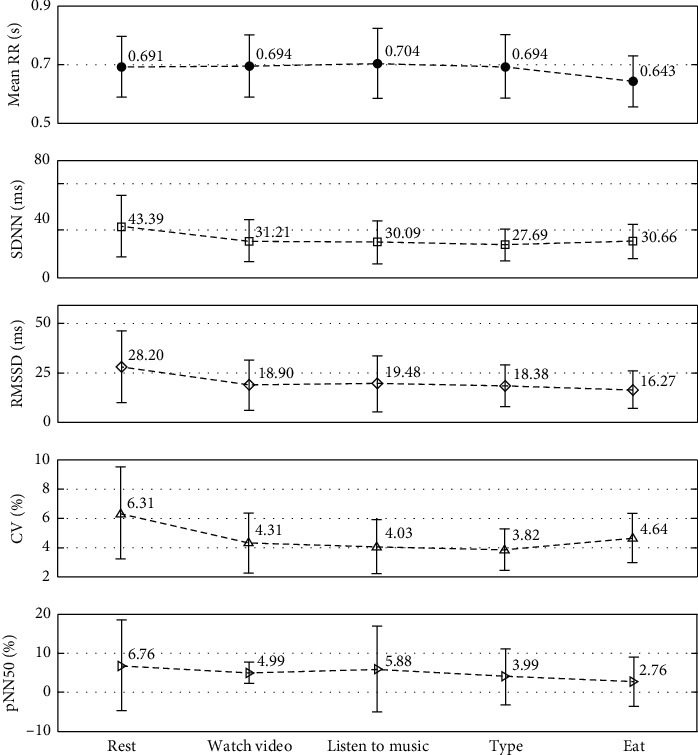
Average HR under different scenarios for the ten subjects.

We analyzed the time domains of the signals captured by our system (HRV_CC-ECG_) and the standard signals captured by the commercial instrument (HRV_Standard_) under different scenarios (resting, video-watching, music-listening, typing, and eating) to obtain the mean RR, SDNN, RMSSD, CV, NN50, and pNN50 parameters, as well as the means and standard deviations of the various HRV_CC-ECG_ and HRV_Standard_ parameters. We then ran *T*-tests on the HRV_CC-ECG_ and HRV_Standard_ parameters to obtain *p* values and correlation coefficients for different scenarios, as shown in Tables 2–6. From these tables, we can see that the Pearson correlation coefficient (*r*) between the HRV_CC-ECG_ and HRV_Standard_ data is close to 1. There were no significant differences between HRV_CC-ECG_ and HRV_Standard_ measurements during resting, video-watching, music-listening, typing, or eating.  Figure 15 is a graph showing the different parameter values for the ten subjects. Since eating tends to cause the heart rate to rise, the standard deviation is smaller for mean RR time intervals in the eating scenarios. The mean SDNN changes for different scenarios and the most significant change in standard deviation was found in the resting scenarios. The same was found for RMSSD, CV, and pNN50, although the standard deviation was found to be the greatest for pNN50 in the music-listening scenario. This could be due to the short intervals between heartbeats when different individuals listened to music.

### 4.5. Long-Term Measurements

**Figure 16:**
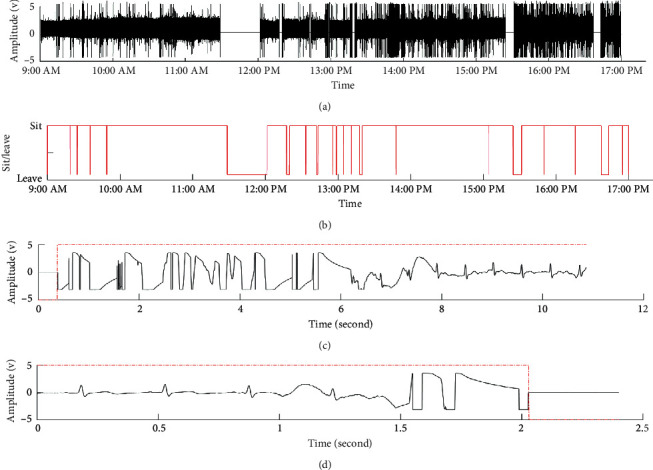
(a) Measurement of ECG. (b) Switch signal. (c) Sitting on the chair. (d) Leaving the chair.

**Figure 17:**
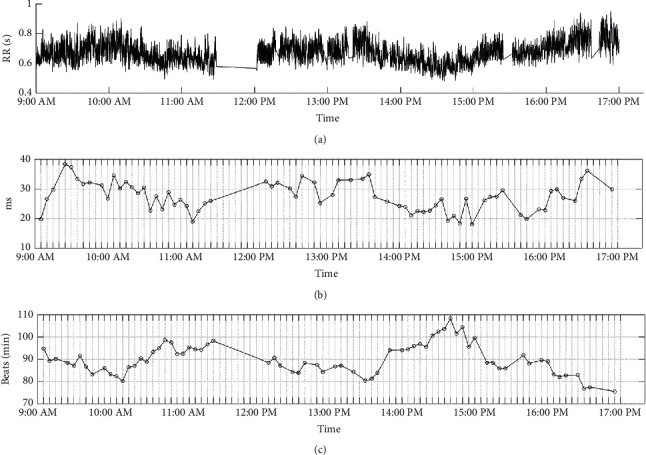
(a) RR time series. (b) SDNN. (c) HR.

Our system is capable of making long-term measurements, a task not easily achieved by contact-type commercial instruments unless subjects carry and stay in contact with the device all the time. Excluding the time period when they left to purchase food, eat, or go to the bathroom, they spent pretty much the whole day doing work at their computers until they left the lab at 5 pm.  Figure 16(a) shows the capacitive ECG signals captured over 8 hours, while Figure 16(b) indicates the switch signals. When the electrical potential is high, this means that the subject is in his seat, whereas low electrical potential indicates that the subject is not in his seat. For example, the electrical potential was low from 11:30–12:04 when the subject left to purchase food. When looking at Figure 16(b), we can reference Figure 17(a) and see that when the subject was not in his seat, no signals were captured.  Figure 16(c) shows changes in ECG capturing every time the subject sat down in his seat. In this instance, the ECG signal only emerged 6 seconds after the subject had sat down.  Figure 16(d) shows the waves captured by the system when the subject was not in his seat. We can see that before the subject left his seat, there was a small period where there were transient waveforms. Additionally, we can see from Figure 16(a) that there was more motion noise in the afternoon compared to the morning. Figure 17(a) shows the R-R time interval curves measured during the 8 hours. Additionally, the SDNN and HR curves obtained every five minutes are shown, respectively, in Figures 17(b) and 17(c).

## 5. Discussion

Our results show that average R-peak detection accuracy Se and PPV for the ten subjects were 0.983 and 0.991, respectively. The Se and PPV of all ten subjects are higher than 0.943 and 0.967, respectively. There were some subjects who had few false positives and false negatives, which showed that it is possible to bring Se and PPV close to 1 if system electrodes have good contact. However, body shape is different for each. Since the same chair back cannot satisfy the needs of all users, there are inevitably gaps between the electrodes and the subject's back, which can cause lower SNR when signals are captured. Therefore, it is important that the electrodes closely match the body curvature. This was particularly obvious in female subjects wearing brassieres. Observation of different scenarios showed that there was more motion noise in dynamic scenarios compared to peaceful scenarios. The accuracy of R-peak detection was also lower.

Friction noise is generated when any of the user's limbs is moved, and thus from the results of the R-R time interval correlations, we found that the accuracy of R-peak detection was lower in the typing and eating scenarios. Even so, erroneous results only made up around 0.5% of all results. After removing erroneous results, a comparison of RR time intervals captured by our system and the commercial instrument showed high consistency between the two sets of data, with correlation coefficient *r* approaching 1 and coefficient of determination *r*^2^ approaching 0.999, which indicates a very high positive correlation. The erroneous results were filtered out manually by researchers in this study, but we hope to include more automated filters in the future to increase the stability of the system.

We captured HRV_CC-ECG_ and HRV_Standard_ signals. Differential analysis results for HRV showed that HRV parameters captured by these two systems under resting, video-watching, music-listening, typing, and eating scenarios all had *p* values exceeding 0.01, meaning that there were no significant differences between the two methods. Furthermore, correlation coefficients were all higher than 0.995. The correlation of NN50 and pNN50 parameters was lower in the typing and eating scenarios, mainly due to the subject movement as the capacitive system capture signals through contact between the skin and electrodes. The filter frequencies used for these two systems were also different, which could have caused other differences during the process of signal capture. Another factor that could have caused a lower correlation was the gaps between the skin and the electrodes due to clothing. This could have affected signal capture, particularly in dynamic scenarios where vertical motions on the electrodes may have caused interference. Past researchers [36] have suspected that noncontact signals and standard ECG signals have the same cyclical signals, and the waves captured are similar to that of ballistocardiogram (BCG) signals with flatter wave peaks, which can also cause errors when capturing R-peaks. This phenomenon was particularly apparent in dynamic scenarios.

In addition to using different scenarios to verify the capabilities of our system compared to commercial systems, we were also able to observe physiological changes in subjects under different scenarios, for example, all subjects showed increases in HR when eating, increasing SDNN when resting, and decreasing SDNN when not resting. These types of physiological phenomena are consistent with the conclusions of past studies [35]. Finally, this study also tested the long-term measurement capabilities of our system. Over the course of 8 hours, we found that motion noise was more prominent in the afternoon compared with the morning. We speculate that the main reason is the noise caused by the tiny movement of the contact surface between the subject and the capacitive electrode and that this tiny movement may be affected by the subject's concentration and emotional fluctuations in the afternoon. Therefore, when monitoring subjects over a long period of time, it is important to consider the comfort of users and the stability of system monitoring, as well as the reliability of signal capturing. To ensure close contact between the electrodes and the body in order to minimize motion noise, our noncontact hardware may have caused discomfort for some subjects. We sought to balance signal quality and subject comfort by using curved electrodes and an ergonomic chair back, but there is still room for improvement with this system. In the future, we may use soft electrodes and curved chair backs for better results. Currently, many wearable devices still cause discomfort to users. Over a 24-hour day where the subject is awake for 16 hours and asleep for 8 hours, the nonperception of this long-term physiological signal measurement system has the potential to be used during sleeping. This noncontact electrode technology can also be used in many settings, for example, in office chairs, sofas, beds, car seats, toilets, and so on. Overall, we hope to conduct health monitoring at home and in the office on subjects dressed in normal clothing, with no need for the system to come into contact with the skin, and with no need for hardware wiring. This does not affect the daily activities of users and can help them overcome physiological and mental stress. In summary, our noncontact, nonbinding, nonperception, and wireless system can facilitate health monitoring over a long period of time without affecting the daily activities of users. During the use of the noncontact seat, each user's sitting posture is different, and the cushion touches the microswitch to start the system when the user is lying and sitting. However, when the user is forward-leaning and sitting, the system will not be activated. Therefore, the system does not capture the heart rate information every time the user sits down. However, as long as the user uses the system for a long time, the system will automatically start the system to obtain the user's heartbeat information, the data will be transmitted to the back-end server for big data collection and analysis, and the system will provide users with long-term health information.

## 6. Conclusions

This study used noncontact active electrodes, along with a home or office computer chair, to develop a “capacitive heart rate monitoring system.” In the simulation study, daily home and office scenarios were used to verify the capabilities of this system as compared with commercial instruments. Our results showed high accuracy in R-peak detection, with mean Se and PPV at 0.983 and 0.991, respectively. The R-R time intervals of both systems were highly consistent. With the correlation coefficient *r* reaching almost 1.000 and differences between the two systems falling within ±10 ms range, the two systems not only show a high positive correlation but also exhibit tiny differences in measurement results. Additionally, the time-domain parameters HRV_CC-ECG_ and HRV_Standard_ show no significant difference under the four different scenarios. Finally, our system was used to conduct measurements over 8 hours and data were transmitted wirelessly to the server in real time. It was demonstrated that the system can achieve the goal of nonperception and is therefore useful for application of heart rate monitoring in daily life.

## Figures and Tables

**Table 1:** Performance of R-peak detection.

Subject	Actual total heartbeat number	Detecting total heartbeat number	True positive (TP)	False positive (FP)	False negative (FN)	Sensitivity (Se)	Positive predictive value (PPV)
1	2727	2725	2723	2	4	0.999	0.999
2	2566	2524	2478	46	88	0.966	0.982
3	3355	3355	3355	0	0	1.000	1.000
4	2577	2569	2555	14	22	0.991	0.995
5	2534	2510	2494	16	40	0.984	0.994
6	3052	3048	3034	14	18	0.994	0.995
7	2248	2247	2246	1	2	0.999	1.000
8	2556	2470	2394	76	162	0.937	0.969
9	3272	3204	3153	51	119	0.964	0.984
10	3657	3646	3636	10	21	0.994	0.997
Average value						0.983	0.991

**Table 2:** Mean, standard deviation, correlation coefficient, and *p* values of HRV_CC-ECG_ and HRV_Standard_ data under the resting scenario.

Parameter	Unit	Average (STD)		*T* value	*p*	Correlation coefficient
		HRV_*cc*_	HRV_Standard_			
Mean RR	(ms)	691.44 (104.44)	691.35 (104.27)	1.753	0.114	1.000
SDNN	(ms)	43.39 (26.36)	45.37 (26.31)	0.594	0.567	1.000
RMSSD	(ms)	28.20 (18.52)	28.32 (18.52)	−1.413	0.191	1.000
CV	(%)	6.31 (3.15)	6.31 (3.14)	0.606	0.559	1.000
NN50		5.09 (8.58)	5.15 (8.68)	−0.887	0.398	1.000
pNN50	(%)	6.76 (11.70)	6.84 (11.84)	−0.893	0.395	1.000

**Table 3:** Mean, standard deviation, correlation coefficient, and *p* values of HRV_CC-ECG_ and HRV_Standard_ data under the video-watching scenario.

Parameter	Unit	Average (STD)		*T* value	*p*	Correlation coefficient
		HRV_*cc*_	HRV_Standard_			
Mean RR	(ms)	694.42 (106.35)	694.33 (106.18)	1.849	0.098	1.000
SDNN	(ms)	31.21 (18.02)	31.20 (18.02)	0.303	0.769	1.000
RMSSD	(ms)	18.90 (12.85)	19.05 (12.87)	−1.688	0.126	1.000
CV	(%)	4.31 (2.05)	4.30 (2.05)	0.317	0.759	1.000
NN50		3.75 (6.15)	3.74 (6.07)	0.148	0.885	0.999
pNN50	(%)	4.99 (2.68)	4.97 (8.34)	0.226	0.826	1.000

**Table 4:** Mean, standard deviation, correlation coefficient, and *p* values of HRV_CC-ECG_ and HRV_Standard_ data under the music-listening scenario.

Parameter	Unit	Average (STD)		*T* value	*p*	Correlation coefficient
		HRV_*cc*_	HRV_Standard_			
Mean RR	(ms)	703.65 (119.47)	703.53 (119.28)	1.990	0.078	1.000
SDNN	(ms)	30.09 (18.37)	30.09 (18.36)	−0.268	0.795	1.000
RMSSD	(ms)	19.48 (14.39)	19.63 (14.42)	−2.464	0.036	1.000
CV	(%)	4.03 (1.84)	4.03 (1.84)	−0.257	0.803	1.000
NN50		4.21 (7.66)	4.36 (7.89)	−1.964	0.081	1.000
pNN50	(%)	5.88 (10.99)	6.09 (11.32)	−1.884	0.092	1.000

**Table 5:** Mean, standard deviation, correlation coefficient, and *p* values of HRV_CC-ECG_ and HRV_Standard_ data under the typing scenario.

Parameter	Unit	Average (STD)		*T* value	*p*	Correlation coefficient
		HRV_*cc*_	HRV_Standard_			
Mean RR	(ms)	693.95 (107.97)	693.86 (107.81)	1.777	0.109	1.000
SDNN	(ms)	27.69 (13.46)	27.71 (13.42)	−0.510	0.623	1.000
RMSSD	(ms)	18.38 (10.86)	18.64 (10.79)	−1.873	0.094	0.999
CV	(%)	3.82 (1.44)	3.82 (1.43)	−0.733	0.482	1.000
NN50		3.01 (5.18)	3.06 (5.74)	−0.208	0.840	0.995
pNN50	(%)	3.99 (7.13)	4.07 (7.92)	−0.254	0.805	0.996

**Table 6:** Mean, standard deviation, correlation coefficient, and *p* values of HRV_CC-ECG_ and HRV_Standard_ data under the eating scenario.

Parameter	Unit	Average(STD)		*T* value	*p*	Correlation coefficient
		HRV_*cc*_	HRV_Standard_			
Mean RR	(ms)	642.87 (87.50)	642.86 (87.36)	0.241	0.815	1.000
SDNN	(ms)	30.66 (14.64)	30.65 (14.63)	0.132	0.898	1.000
RMSSD	(ms)	16.27 (9.64)	16.53 (9.59)	−1.723	0.119	0.999
CV	(%)	4.64 (1.68)	4.64 (1.67)	0.034	0.974	1.000
NN50		2.30 (5.08)	2.27 (5.00)	1.000	0.343	0.996
pNN50	(%)	2.76 (6.28)	2.73 (6.18)	1.000	0.342	0.996

## Data Availability

The data used to support the findings of this study are currently under embargo while the research findings are commercialized. The data can be available after the whole research is commercialized, and requests for data will be considered by the corresponding author.
